# Anterior Circulation Fusiform Aneurysms Have a Lower Occlusion Rate After Pipeline Embolization Device Treatment Than Posterior Circulation Fusiform Aneurysms: A Multicenter Cohort Study

**DOI:** 10.3389/fneur.2022.925115

**Published:** 2022-06-02

**Authors:** Chao Xu, Pei Wu, Liang Zou, Shancai Xu, Bin Luo, Xinjian Yang, Huaizhang Shi

**Affiliations:** ^1^Department of Neurosurgery, First Affiliated Hospital of Harbin Medical University, Harbin, China; ^2^Beijing Neurosurgical Institute, Beijing Tiantan Hospital, Capital Medical University, Beijing, China; ^3^Department of Interventional Neuroradiology, Beijing Neurosurgical Institute and Beijing Tiantan Hospital, Capital Medical University, Beijing, China

**Keywords:** fusiform aneurysm, pipeline embolization device, anterior circulation, posterior circulation, flow diverter devices

## Abstract

**Objective:**

Intracranial fusiform aneurysms are uncommon and can occur in vessels of the anterior circulation (AC) or posterior circulation (PC). While flow diversion is one treatment option, research into Pipeline Embolization Device (PED) treatment is lacking. This study explored the efficacy and safety of PED treatment for intracranial fusiform aneurysms, and compared therapeutic effects between AC and PC aneurysms.

**Methods:**

In the post-market multi-center cohort study of embolization of intracranial aneurysms with PED in China (PLUS) registry study, we retrospectively analyzed 71 fusiform aneurysms in 67 patients among 1,171 patients treated with a PED from November 2014 to October 2019. The general characteristics, perioperative status, aneurysm occlusion rate at the last follow-up angiography, and changes in modified Rankin Scale scores were analyzed. Aneurysms were divided into AC and PC groups, and univariate and multivariate analyses were conducted.

**Results:**

The study included 26 AC (25 patients) and 45 PC (42 patients) aneurysms. A total of 75 PEDs were used, an average of 1.1 PEDs were used, and the median follow-up was 6.7 months. Fifty aneurysms (71.4%) were occluded and twenty (28.5%) were incompletely occluded. There were significantly more occluded aneurysms in the PC group than in the AC group (12 vs. 38; *P* = 0.001). Risk factors for incomplete occlusion were AC aneurysms (*P* = 0.001) and a perforating artery originating from the aneurysm (*P* = 0.006). The mean modified Rankin Scale score was significantly lower at the last follow up than preoperatively (0.58 vs. 0.21; *P* = 0.0001).

**Conclusion:**

Non-overlapping PED is a safe and effective treatment for both AC and PC fusiform aneurysms. The occlusion rate of AC fusiform aneurysms is lower than that of PC.

## Introduction

Fusiform intracranial aneurysms are rare, and their unique morphology and pathological development continue to present a challenge for neurosurgical treatment (1–4). While the Pipeline Embolization Device (PED; Medtronic, Minneapolis, MN, USA) provides a treatment option for fusiform aneurysms, few studies have investigated this (5–10), especially in posterior circulation (PC) and anterior circulation (AC) fusiform aneurysms beyond the circle of Willis, for which PED use is off-label (11). Previous studies on fusiform aneurysms have mostly been single-center studies; the present study included data from multiple centers. To summarize, this post-market multi-center cohort study of embolization of intracranial aneurysms with PED in China (PLUS) registry study investigated the clinical prognosis and occlusion rate of intracranial fusiform aneurysms treated with a PED, and the efficacy of PEDs in the treatment of AC and PC intracranial fusiform aneurysms.

## Materials and Methods

### Data Collection and Follow-Up

In this consecutive, real-world cohort registry study, we retrospectively collected data on aneurysms treated with PEDs from a database of 14 participating Chinese institutions, between November 2014 and October 2019. The local institutional review boards and ethics committees approved the study and the use of patients' data. All operations were performed with patients' written informed consent.

Fusiform aneurysms were defined as aneurysmal dilatations of >50% of the vessel wall circumference without a discrete aneurysm neck (2, 10). Aneurysms were categorized as fusiform based on 3D rotational digital subtraction angiography (DSA) findings, and all cases were reviewed for inclusion by the senior author. Purely saccular aneurysms, blister aneurysms, sidewall aneurysms (aneurysms located in the lateral wall artery without involvement of the entire artery), and dissecting aneurysms (retention of contrast in the aneurysm during angiography) were excluded. The AC group and PC group were defined according to the aneurysm location.

The collected data included patients' demographic characteristics, aneurysm characteristics (side, location, clinical manifestations, preoperative modified Rankin Scale (mRS) score, diameter of parent artery, maximum diameter of aneurysm, recurrent aneurysm), and treatment status (size of PED, number of PEDs, PED + coils, operation related complications, post-operative O'Kelly–Marotta (OKM) grading scale (12)). Data from branch arteries originating from fusiform aneurysms, such as the posterior communicating artery, ophthalmic artery, anterior inferior cerebellar artery, and posterior inferior cerebellar artery, were also collected. Given that fusiform aneurysms do not have a distinct aneurysm neck, the contrast is obviously retained in the entire aneurysm after PED implantation; thus, we used the retention time of the contrast as the main basis for OKM classification. Data on the follow-up status (months of follow-up, aneurysm occlusion at the last radiogram follow-up, and mRS score at the last follow-up) were also collected. Aneurysms were divided into four categories according to maximum diameter, namely, ≤ 7 mm, 7–15 mm, 15–23 mm, and > 23mm.

Follow-up DSA was performed 6 months after operation and repeated again between 12 and 36 months. Aneurysm occlusion was classified using the OKM grading scale.

### Perioperative Management

All patients received an antiplatelet regimen that included aspirin 100 mg/day or 300 mg/day and clopidogrel 75 mg/day for 5 days prior to the operation. Patients who were identified as clopidogrel non-responders received aspirin 100 mg/day and ticagrelor 90 mg twice daily. All patients demonstrated optimal platelet activity suppression before PED placement. Board-certified neuro-endovascular surgeons performed all procedures. Intravenous heparin was administered intra-procedurally to achieve an activated clotting time of >250 s. Heparin was discontinued after completing the procedure. A continuous dual antiplatelet therapy regimen was applied after PED placement, including aspirin 100 mg/day, clopidogrel 75 mg/day, or ticagrelor 90 mg/day for 6 months. After 6 months, the decision to stop clopidogrel or ticagrelor was made according to angiography results. Aspirin 100 mg/day was recommended to be taken for life.

### Statistical Analysis

Categorical variables are reported as proportions, and continuous variables are reported as the median and quartile or range. The Chi-square test, Mann–Whitney U, and Wilcoxon rank sum test were used to compare variables between the two groups. Univariate analysis was performed for age, sex, hypertension, smoking status, aneurysm characteristics, treatment status, and follow-up status in relation to incomplete occlusion of the aneurysm. The predictive factors (*P* < 0.10) identified in the univariate analysis, as well as treatment method, postoperative OKM grading scale, and last radiographic follow-up time, were included in the multivariate analysis. *P*-values < 0.05 were considered statistically significant. Statistical analysis was performed using SPSS 25 (IBM Corp., Armonk, NY, USA).

## Results

### Patient and Aneurysm Characteristics

A total of 71 fusiform aneurysms in 67 patients were retrospectively identified. Twenty-five patients (37.3%) were female and 42 patients (62.7%) were male (median age 51 years, range 8–75 years). There were 32 aneurysms on the left side (45.1%) and 39 on the right side (54.9%). Twenty-six aneurysms (36.6%) were located in the AC and 45 aneurysms (63.4%) in the PC (Table 1). Two aneurysms (3%) were recurrent and had undergone prior endovascular treatment. A total of 18 patients were asymptomatic (26.9%), 35 patients (52.2%) had headache and dizziness, 10 patients (14.9%) had neurological symptoms, including dysarthria in two, oculomotor nerve palsy in one, visual field defect in two, weak limb power in two, and limb numbness in three, and 4 patients (6%) had subarachnoid hemorrhage. Thirty-seven cases (55.2%) had a history of smoking. The median diameter (interquartile range) of the parent artery was 3.5 mm (2.9–4.17 mm). A total of 71 aneurysms were divided into the ≤ 7 mm group (18 aneurysms), 7–15 mm group (37 aneurysms), 15–23 mm group (10 aneurysms), and > 23 mm group (6 aneurysms). In the 67 patients, a total of 75 PEDs were used; 7 patients had two PED implants, one of the other 60 patients had PED implanted in his bilateral internal carotid arteries. An average of 1.1 PEDs were used. Application PED size median was 4.0 mm (3.5–4.25 mm). Among the aneurysms, 52 aneurysms (73.2%) were treated with PED only, and 19 aneurysms (26.8%) were treated with PED + coils (Tables 2, 3).

**Table 1:** Location of the treated aneurysms.

**AC (*n* = 26), n (%)**		**PC (*n* = 45) *n* (%)**
ICA cavernous	9 (34.6%)	V4	37 (82.2%)
ICA ophthalmic	7 (26.9%)	Vertebral-basilar	5 (11.1%)
ICA Pcom	6 (23.1%)	BA	2 (4.4%)
MCA	4 (15.4%)	PCA	1 (2.2%)

*AC, anterior circulation; PC, posterior circulation; ICA, internal carotid artery; MCA, middle cerebral artery; PCA, posterior cerebral artery; Pcom, posterior communicating; BA, basilar artery*.

**Table 2:** Baseline characteristics of 67 patients with 71 fusiform aneurysms.

**Characteristics**	**Total**	**AC**	**PC**	***P-*value**
No. of patients	*n* = 67	25	42	
No. of aneurysms	*n* = 71	26 (36.6%)	45 (63.4%)	
Median age in years (range)	51 (8–75)	53 (14–75)	51 (8–69)	0.817
Sex				0.015
Male	42 (62.7%)	11 (44%)	31 (73.8%)	
Female	25 (37.3%)	14 (56%)	11 (26.2%)	
Smoking	37 (55.2%)	10 (40%)	27 (64.3%)	0.053
Side				0.178
Left	32 (45.1%)	9 (34.6%)	23 (51.1%)	
Right	39 (54.9%)	17 (65.4%)	22 (48.9%)	
Presenting symptoms				
Asymptomatic	18 (26.9%)	10 (40.0%)	8 (19.0%)	0.061
Headache/dizziness	35 (52.2%)	11 (44.0%)	24 (57.1%)	0.298
Neurological deficit	10 (14.9%)	2 (8.0%)	8 (19.0%)	0.223
SAH				0.591
Yes < 2weeks	4 (6.0%)	2 (7.7%)	2 (4.8%)	
No	63 (94.0%)	23 (92.0%)	40 (95.2%)	
Pretreatment mRS score				0.709
0–2	65 (97.0%)	24 (96.0%)	41 (97.6%)	
3–5	2 (3.0%)	1 (4.0%)	1 (2.4%)	
Parent artery (median; IQR) (mm)	3.5 (2.9–4.1)	3.7 (3.1–4.2)	3.2 (2.8–3.9)	0.04
Maximal diameter				0.553
≤ 7 mm	18 (25.4%)	7 (26.9%)	11 (24.4%)	
7–15 mm	37 (52.1%)	11 (42.3%)	26 (57.8%)	
15–23 mm	10 (14.1%)	5 (19.2%)	5 (11.1%)	
> 23 mm	6 (8.5%)	3 (11.5%)	3 (6.7%)	
Artery from aneurysms	19 (26.8%)	8 (30.8%)	11 (24.4%)	0.562
Previous treatment				
Endovascular	2 (3.0%)	1 (3.8%)	1 (2.4%)	0.725

*AC, anterior circulation; PC, posterior circulation; IQR, Interquartile range*.

**Table 3:** Outcome measures of 67 patients with 71 fusiform aneurysms.

**Characteristics**	**Total**	**AC**	**PC**	***P*-value**
No. of PEDs	75	28	47	0.792
Double PED	7 (10.3%)	3 (12.0%)	4 (9.3%)	0.792
Average number of PED (range)	1.10 (1–2)	1.12 (1–2)	1.09 (1–2)	0.726
Treatment				0.562
PED only	52 (73.2%)	18 (69.2%)	34 (75.6%)	
PED + coil	19 (26.8%)	8 (30.8%)	11 (24.4%)	
Size of PED mm (median; IQR)	4.0 (3.5–4.25)	4.0 (3.5–4.5)	4.0 (3.5–4.25)	0.451
Length of PED mm (median; IQR)	30 (25–35)	30 (25–35)	30 (25–35)	0.906
Complications				
Thromboembolic	0	0	0	
Hemorrhagic	3 (4.4%)	2 (8.0%)	1 (2.3%)	0.275
Ischemia	3 (4.4%)	1 (4.0%)	2 (4.7%)	0.900
Postoperative angiography				1.000
OKM A-B-C 3	39 (54.9%)	14 (53.8%)	25 (55.6%)	
OKM A-B-C 2	17 (23.9%)	7 (26.9%)	10 (22.2%)	
OKM A-C 1	15 (21.1%)	5 (19.2%)	10 (22.2%)	
LRF (m) median (range)	6.7 (3–36.2)	6.4 (3.6–26.8)	7.2 (3–36.2)	0.628
Follow-up occlusion rate				0.001
OKM-D	50 (71.4%)	12 (48%)	38 (84.4%)	
OKM-B2	15 (21.4%)	9 (36%)	6 (13.3%)	
OKM-B3	5 (7.1%)	4 (16%)	1 (2.2%)	
mRS at last follow-up				0.065
0–2	65 (97.0%)	23 (92.0%)	42 (100%)	
3–5	1 (1.4%)	1 (4.0%)	0	
6-death Mortality w/in ≤ 30 days	1 (1.4%)	1 (4.0%)		

*AC, anterior circulation; PC, posterior circulation; PED, pipeline embolization device; IQR, Interquartile range; OKM, O'Kelly–Marotta grading scale; LRF, last radiographic follow-up; m, months; IMF, Imaging modality at follow-up; DSA, digital subtraction angiography; CTA, computed tomography angiography; mRS, modified Rankin Scale*.

### Clinical and Imaging Results

Angiography at the final follow up (median, 6.7 months; range, 3–36.2) demonstrated OKM-D type occlusion (no filling) in 50 aneurysms (71.4%), as complete obliteration; incomplete aneurysm occlusion occurred in 20 aneurysms; of these, 15 were OKM-B2 (subtotal filling), and 5 were OKM-B3. In the AC aneurysms, 12 (48%) were OKM-D, 9 (36%) were OKM-B2, and 4 (16%) were OKM-B3. In the PC aneurysms, 38 (84.4%) were OKM-D, 6 (13.3%) were OKM-B2, and 1 (2.2%) was OKM-B3 (Table 3). Among the 50 complete obliteration aneurysms, nine aneurysms with branch arteries originated from the aneurysms, accounting for 50% of this type of aneurysms compared with the other 41 (78.8%) aneurysms (*P* = 0.02).

### Multivariate Analysis

**Figure 1:**
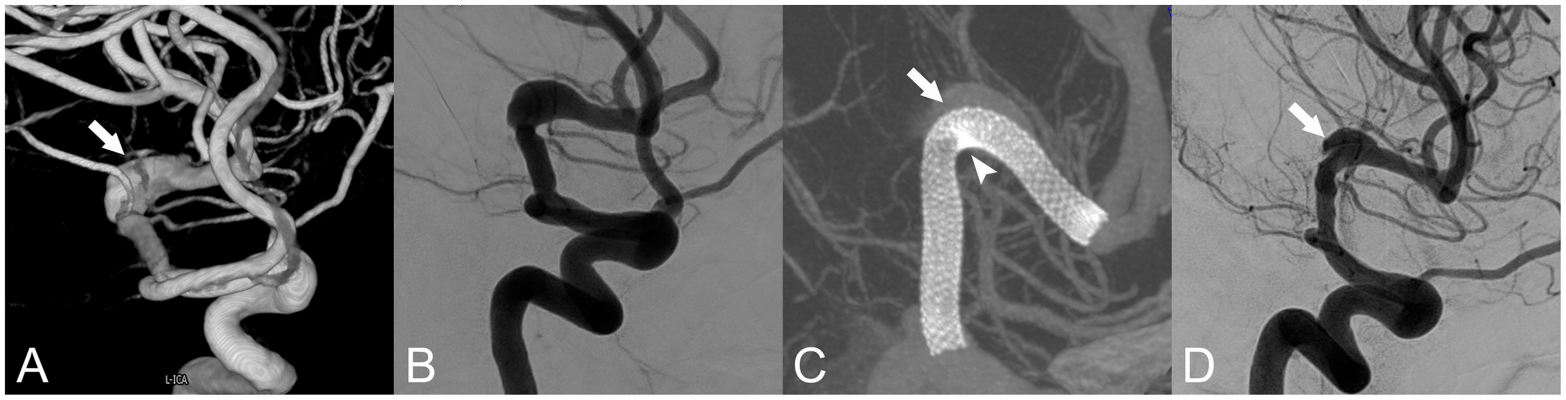
Fusiform aneurysm of the left middle cerebral artery. **(A)** Three-dimensional reconstruction of the fusiform aneurysm (white arrow). **(B)** Anteroposterior projection by digital subtraction angiography (DSA). **(C)** Vaso CT indicated that the metal coverage of the small curve (arrow head) was higher than that of the large curve (white arrow). **(D)** A 9-month follow-up angiography showed that the aneurysm remained residual (white arrow), and the intima had formed between the stent and the aneurysm.

**Figure 2:**
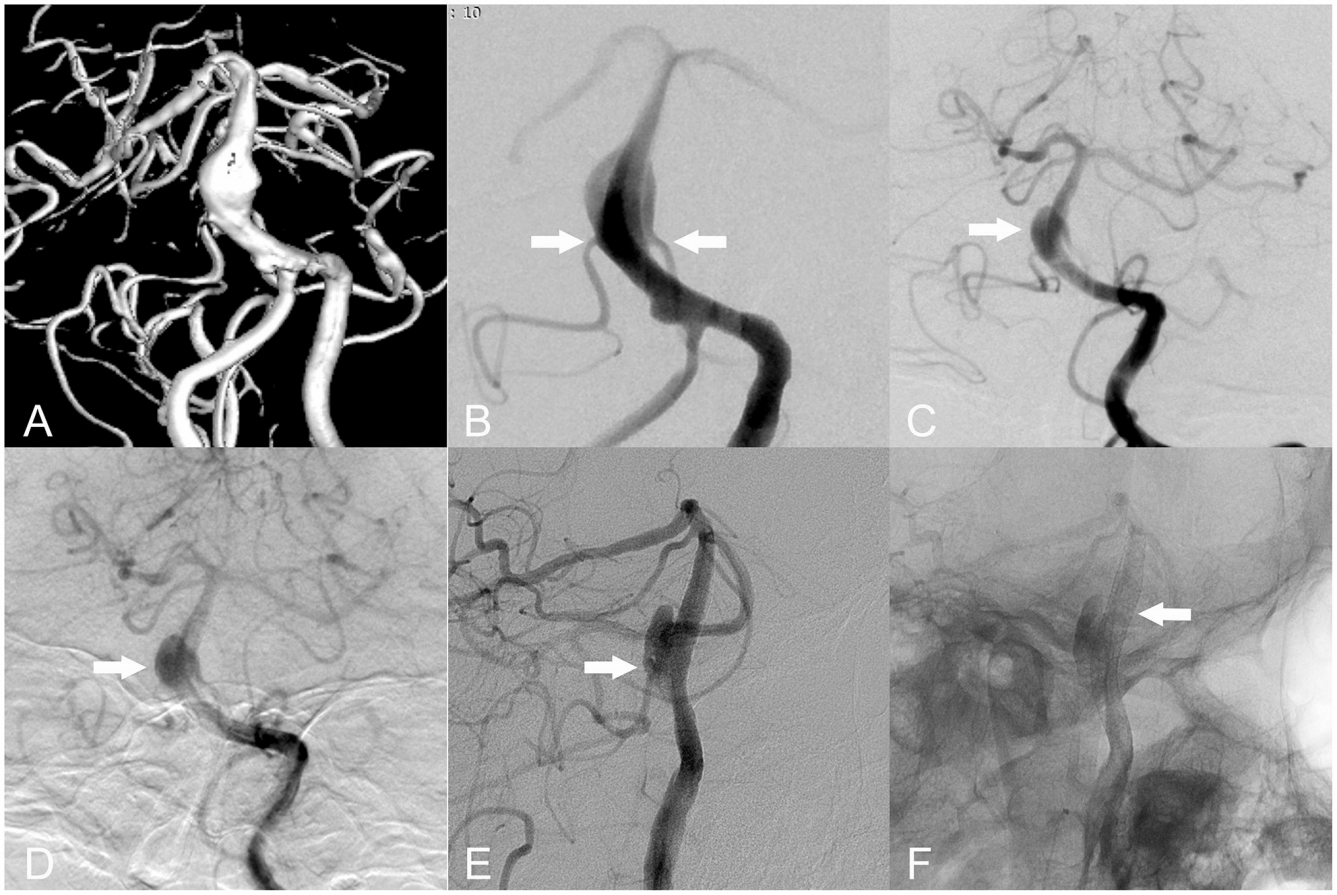
Basilar fusiform aneurysms. **(A,B)** Bilateral anterior inferior cerebellar arteries (AICAs) originate from the aneurysmal body (white arrow). **(C,D)** A 9-month follow-up angiography revealed residual aneurysm. **(E)** Blood flow through aneurysmal bodies supplied blood to the AICA (white arrow). **(F)** A 9-month follow-up angiography revealed no intra stent stenosis (white arrow).

The multivariate analysis revealed that aneurysms located in the AC (odds ratio 8.979, 95% confidence interval 2.337–34.504; *P* = 0.001) and perforating artery originating from the aneurysm (odds ratio 8.655, 95% confidence interval 1.830–40.923; *P* = 0.006) were risk factors for incomplete occlusion (Figures 1, 2; Table 4).

**Table 4:** Predictors of incomplete occlusion of fusiform aneurysms treated with PEDs.

**Parameter**	Univariate analysis^*^		**Multivariate analysis**	
	**OR (95% CI)**	***P-*value**	**OR (95% CI)**	***P*-value**
AC vs. PC	5.881 (1.909–18.114)	0.002	8.979 (2.337–34.504)	0.001
PED-only vs. PED + coils	2.667 (0.682–10.428)	0.159	4.133 (0.760–22.471)	0.101
Artery from aneurysms	3.727 (1.194–11.639)	0.024	8.655 (1.830–40.923)	0.006
OKM1,2 vs. OKM 3	2.250 (0.781–6.485)	0.133	2.782 (0.695–11.133)	0.148
LRF (m) <6.7	0.755 (0.267–2.139)	0.597	0.465 (0.122–1.769)	0.261

*AC, anterior circulation; PC, posterior circulation; PED, pipeline embolization device; OKM, O'Kelly–Marotta grading scale; LRF, last radiographic follow-up; m, month*.

* *Also entered in the univariate analysis but not significant: sex, smoking, presenting symptoms, maximum aneurysm diameter, aneurysm neck, number of PEDs*.

### Clinical Outcomes

**Figure 3:**
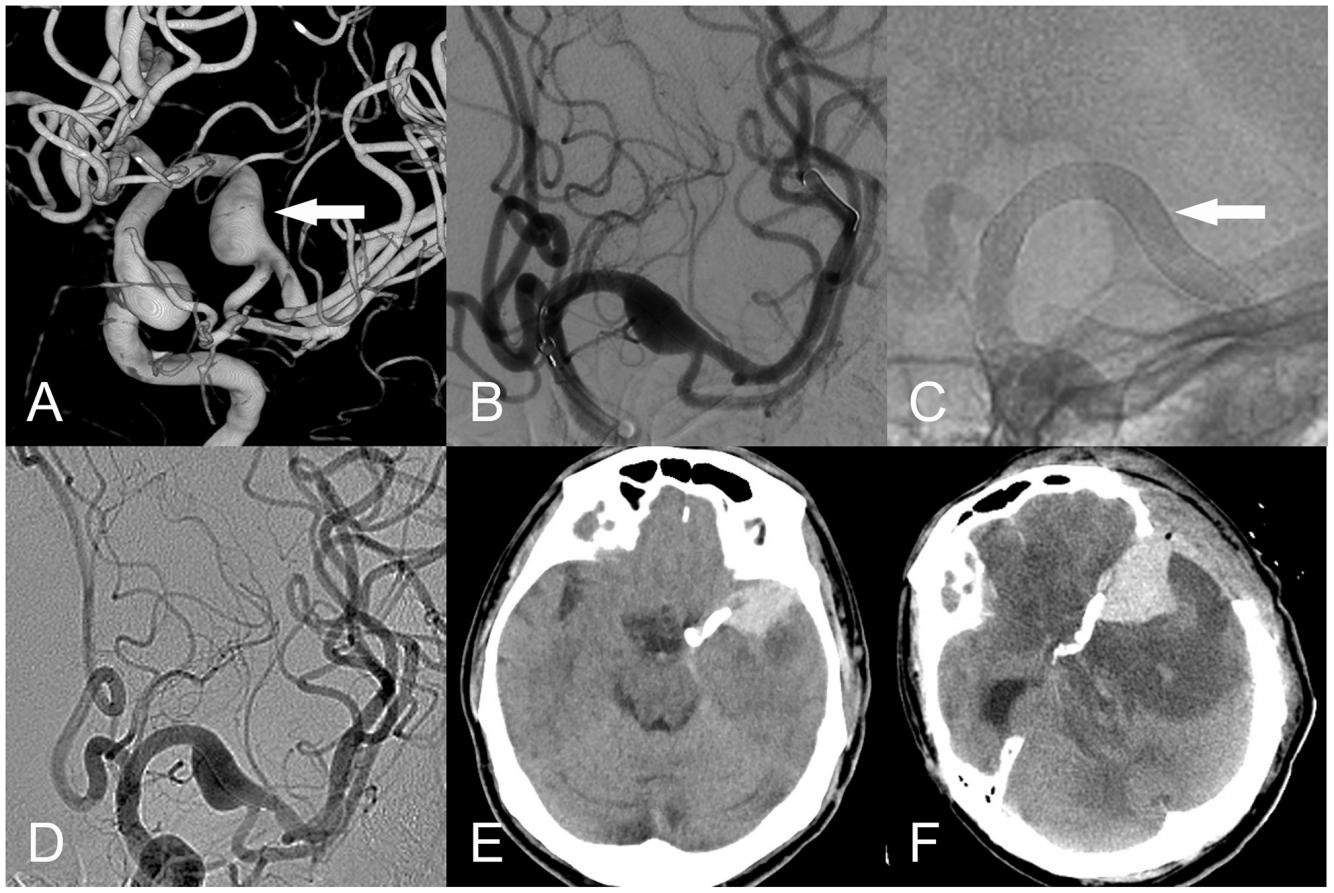
Fusiform aneurysm of the left middle cerebral artery. **(A)** Three-dimensional reconstruction (white arrow). **(B)** The PED was implanted intraoperatively uneventfully (white arrow). **(C,D)** The PED was well adherent, and the contrast was obtained after the release of the PED. **(E)** Nine hours postoperatively, the patient developed severe headache. CT suggested cerebral hemorrhage with subarachnoid hemorrhage, and decompressive bone flap was performed urgently. **(F)** 1 day after decompressive surgery by deparaffinization flap, CT suggested significant brain edema around the intracerebral hemorrhage.

The mRS score was improved at the last follow-up compared with the preoperative score in 24 (35.8%) patients, was unchanged in 42 (62.7%) patients, and 1 patient died due to cerebral hemorrhage postoperatively (Figure 3). The mean preoperative mRS score (standard deviation) was 0.58 (0.89), and the mean mRS score at the last follow-up was 0.21 (0.89). This difference was significant (*P* = 0.0001; Tables 5, 6).

**Table 5:** Clinical status of included patients.

**mRS**	**Improved**	**Unchanged**	**Worse**	***P*-value**
Total (*n* = 67), *n* (%)	24 (35.8)	42 (62.7)	1 (1.5)	0.0001
AC (*n* = 25), *n* (%)	7 (28.0)	17 (68.0)	1 (4.0)	0.229
PC (*n* = 42), *n* (%)	17 (40.5)	25 (59.5)	0 (0)	

*AC, anterior circulation; PC, posterior circulation*.

**Table 6:** Flow diversion (PEDs) for treatment of fusiform aneurysms.

		**No. of fusiform**	**Mean**	**Follow up**	**Occlusion**	**Morbidity**	**Mortality**	**Mean mRS score**	**Mean mRS score**
**Author**	**Location**	**aneurysms**	**PED (range)**	**(months)**	**rates *n*%**	***n*%**	***n*%**	**pretreatment**	**at last follow-up**
Siddiqui	PC	7	6.4 (3–9)	NA	29	14	57	2.6	3.3
et al. (5)
Munich	PC	12	3.4 (1–6)	11	90	25	8.3	1.0	1.9
et al. (8)
Monteith	B	24	1.8 (1–6)	6.3	59	16.7	4.2	0.7	1.2
et al. (7)
Natarajan	PC	12	1.7 (1–4)	12–43	100	8.3	0	NA	NA
et al. (9)
Griffin	B	30	1.6 (1–6)	17.4	76	6.7	3.4	NA	NA
et al. (10)
Present	B	71	1.1 (1–2)	6.7	71.4	8.8	1.4	0.58	0.21
study

*Location, anterior or posterior circulation; PC, posterior circulation; B, both circulations; NA, not applicable*.

The postoperative morbidity rate of patients was 8.8%, and the mortality rate was 1.4%. Postoperative complications included intracranial hemorrhage in 3 (4.4%) patients (2 located in the AC and 1 in the PC), ischemia in 3 (4.4%) patients (1 in the AC and 2 in the PC), 1 patient died due to cerebral hemorrhage postoperatively (Figure 3); 2 of the remaining 5 patients had improved mRS scores at the last follow-up compared with the preoperative score, and 3 patients had unchanged mRS scores (Table 3).

## Discussion

Fusiform aneurysms account for 3–13% of all intracranial aneurysms; AC fusiform aneurysms are rare and usually located in the internal carotid artery and middle cerebral artery, and PC fusiform aneurysms are usually located in the vertebral artery and basilar artery (1). About 70% of patients with PC aneurysms are male, and these aneurysms most commonly present as PC ischemic stroke (3). In this study, AC aneurysms accounted for 38% of all treated aneurysms, and most patients were asymptomatic. We found that 62% of fusiform aneurysms were located in the PC, 73% of cases were of male patients, and 61% of patients had headache, dizziness, and ischemia. This retrospective study assessed the safety and efficacy of PED treatment for intracranial fusiform aneurysms, and is the first to compare PED treatment between AC and PC fusiform aneurysms.

### Occlusion of Intracranial Fusiform Aneurysms

The morphology of fusiform aneurysms is a predictor of incomplete occlusion after flow diverter treatment (13). Previous reports of intracranial fusiform aneurysms treated with flow diverter devices (FDD) have been based on small series studies, with an overall occlusion rate of 59–100% (7–10). In one meta-analysis of AC fusiform aneurysms located distal to the circle of Willis and PC fusiform aneurysms, Cagnaggo et al. reported an occlusion rate of 83% (11). Monteith et al. reported 24 intracranial fusiform aneurysms treated with PEDs, 22 of which were followed up with DSA for an average of 6.3 months, with an occlusion rate of 59% (7). Griffin et al. reported 25 cases followed up with DSA for an average of 17.4 months with an occlusion rate of 76% (10). Fischer et al. reported that PED was used to treat 67 fusiform and dissecting aneurysms, and the complete occlusion rate was 67% at the last follow-up of 27.4 months (6). The increase in follow-up time did not increase the aneurysm occlusion rate in those studies. The present multivariate analysis results are consistent with those previous findings, whereby the overall aneurysm occlusion rate was 71.4% at an average follow-up of 6.7 months. In our study, 18 aneurysms with perforating artery originating from the aneurysm, the occlusion rate was 50 vs. 78.8% of the other fusiform aneurysms at an average follow-up of 6.7 months. The difference was statistically significant. In previous studies of saccular aneurysms, the perforating artery originating from the aneurysm was an independent risk factor for incomplete aneurysm occlusion (14–16); for these aneurysms, after PED implantation, the perforating artery still has a sufficient pressure gradient to maintain forward blood flow. This means that sufficient blood flow will be retained in the aneurysm to prevent thrombosis, resulting in an aneurysm that is difficult to occlude. This conclusion applies equally to fusiform aneurysms treated with the PED, and was supported by the present multivariate analysis of intracranial fusiform aneurysms with incomplete occlusion.

The average number of PEDs used to treat AC and PC fusiform aneurysms in this study was 1.12 and 1.09, respectively, which is lower than that reported in previous studies (5, 7–10). The PED landing zone for fusiform aneurysms is just proximal and distal to the lesion. This means that it is theoretically possible to push and pull the PED mesh to increase the metal coverage of the lesion area and increase the flow guidance effect to promote aneurysm healing. Furthermore, given that the PED at the lesion is in a suspended state, especially for lesions with a large curvature, the push and pull technique is less able to achieve the above purpose (6). Multiple PED treatment is a feasible method. Each overlapping PED can increase the metal coverage by 5% and reduce the blood flow velocity in the aneurysm by 30% (6, 10). However, this also increases the complexity and duration of the operation. It can also lead to occlusion of perforating vessels and stenosis of the parent artery (6, 10). Compared with multiple PED treatment, we recommend PED with adjunctive coil treatment. Considering that there is no effective support for PEDs used to treat fusiform aneurysm lesions, adjunctive coils can act as scaffold to reduce foreshortening of the flow diverter and to promote the formation of thrombosis in the aneurysm, leading to occlusion of the aneurysm (9, 10, 17). A PED with coils is recommended for fusiform aneurysms without involvement of the branch vessels in the AC. Compared with AC fusiform aneurysms, PC aneurysms have more perforating vessels, and the use of adjunctive coils for some selected patients has been recommended (18). However, in our study, the PED + coils cases accounted for 30.8% in the AC group and 24.4% in the PC group; the occlusion rate was thus lower in the AC group. Multivariate regression revealed that the use of a PED with adjunctive coils was not associated with aneurysm occlusion. This may be related to loosely packing coils. The degree of loose packing therefore needs to be explored in future work.

### Different Occlusion Rate Between AC and PC Fusiform Aneurysms

Previous studies of small sample size PC fusiform aneurysms have reported occlusion rates in the range of 29–100% (5, 8, 9). In a study by Griessenauer et al. of 131 PC aneurysms with a median follow-up time of 9 months, the occlusion rate of 53 fusiform aneurysms was 59.7% (19). In a recent study by Griessenauer et al. in 149 PC aneurysms, including 42 fusiform aneurysms, with a median follow-up time of 12 months, the occlusion rate was 91.3% (20). Bhogal and colleagues reported 56 PC non-saccular aneurysms, including 24 fusiform aneurysms, with 75% aneurysm occlusion at the last follow-up of 25.2 months (21). There were 45 PC fusiform aneurysms in our study. The median follow-up time was 6.7 months, and the occlusion rate was 84.4%. The occlusion rate of the AC group was 48%. Szikora et al.'s (22) autopsy pathology concluded that AC and PC fusiform aneurysms belong to the same pathological type; according to this conclusion, there should be no significant difference in the occlusion rate of PED treatment. However, we found a significant between-group difference in the occlusion rate. Our multivariate analysis revealed that an aneurysm located in AC was an independent risk factor for incomplete aneurysm occlusion.

In the present study, most AC lesions were located in the cavernous sinus and ocular segments, and most PC lesions in the straight part of the V4 segment. It is easier to use the push-pull technique during PED treatment to form a higher metal coverage at the aneurysm lesion and promote the occlusion of the aneurysm, especially in the straight part of the V4 segment. To adapt to the large curvature of the AC fusiform aneurysm, the metal coverage of the PED in the aneurysm is significantly different between the inner curve and the outer curve (Figure 1), and the blood flow guiding effect is worse than that of a straight blood vessel (23, 24). This could explain the low occlusion rate of AC fusiform aneurysms. Hemodynamically, peak systolic flow velocity values were 30–50% higher in the AC than in the PC (25). Given that flow velocity is correlated with wall shear stress variation (which is tightly linked to aneurysm enlargement, intra-aneurysmal thrombosis, and endothelialization of the parent artery), this difference would become apparent after implantation of the PED, resulting in a differential rate of fusiform aneurysms occlusion between the AC and PC; this has not been specifically studied, but is worth exploring (26–29).

### Complications of Intracranial Fusiform Aneurysms

Previous retrospective studies have reported neurological morbidity and mortality rates of intracranial fusiform aneurysms treated with FDD of 17 and 8%, respectively, (6, 11). Monteith et al. reported a complications rate and mortality rate of 16.7 and 4.2%, respectively, in 24 intracranial fusiform aneurysms treated with PEDs (7). Griffin et al. reported 30 cases of intracranial fusiform aneurysms with a complication rate and mortality rate of 6.7 and 3.4%, respectively, (10). The overall neurological morbidity rate and mortality rate in this study were 8.8 and 1.4%, respectively. Although the mortality rate was lower, the neurological morbidity rate was higher than that reported by Griffin et al. (10). The neurological morbidity rate was not significantly different between the AC and PC aneurysms, at 12 and 7%, respectively.

### Complications of AC Fusiform Aneurysms

The AC neurological morbidity and mortality rates in this group were 12 and 4%, respectively, which were lower than the complication rate of 14% in AC non-saccular aneurysms in the meta-analysis of Cagnaggo et al. (11). Postoperative thrombosis and hemorrhage of fusiform aneurysms are critical factors leading to death. Szikora et al. (22) analyzed the pathology of fusiform aneurysms in patients that died after FDD treatment. They found that the endothelialization of FDD inside the aneurysm may take 12 months or more, which can lead to the two following situations: (1) If the antiplatelet effect is insufficient, thrombotic complications are more likely to develop; (2) Due to the lack of infiltration of vascular smooth muscle cells, FDD cannot undergo endothelialization, and stagnant blood in the aneurysm sac may activate matrix metalloproteinases to break down the aneurysm wall and cause aneurysm rupture (30, 31). This mechanism could have occurred in this group of postoperative cerebral hemorrhages, which caused death in the present study (Figure 3).

### Complications of PC Fusiform Aneurysms

In this study, the neurological morbidity rate in the PC group was 7%, and no patients died. In previous studies, the neurological morbidity rate of PC fusiform aneurysms was 8.3–25%, and the mortality rate was 0–57% (5, 8, 9). These previous results were from studies with small sample sizes. Recently, Bhogal and colleagues reported 56 cases of PC non-saccular aneurysms with a neurological complication rate and mortality rate of 15.5 and 15.5%, respectively, (21). Among these, 24 had fusiform aneurysms, with a postoperative mortality rate of 3.6%. Lopes and colleagues reported that among the 95 PC aneurysms treated with PED, 28 fusiform aneurysms had a neurological complication rate of 19.2% and a mortality rate of 11.5%, and Cox regression analysis revealed that more than 3 PEDs was a related risk factor (32). In several previous studies, the average number of PEDs used was more than 3 (5, 9, 21, 22, 32); the average number of PEDs used in this study was 1.09 in the PC, and the complication and mortality rates were the lowest in this group. The overlapping application of multiple PEDs increases the risk of perforating vessel occlusion caused by thrombosis. Therefore, the minimum number of PEDs required to cover the lesion should be used when treating post-circulating fusiform aneurysms.

### Clinical Outcomes

In our series, the last follow-up mRS score had decreased compared with preoperative scores in 24 patients (35.8%), no change was seen in 42 patients, and 1 patient died. There was a significant difference between the mRS score at the last follow-up and the preoperative mRS score, which indicates that PED therapy was effective in improving patient outcomes. This was also demonstrated in patients who had postoperative complications. Postoperative complications included intracranial hemorrhage in 3 (4.4%) patients, ischemia in 3 (4.4%) patients, and death in 1 patient due to cerebral hemorrhage postoperatively; 2 of the remaining 5 patients had decreased mRS scores at the last follow-up compared with the preoperative score, and 3 patients had unchanged mRS scores of 0. There was no significant difference in the change of mRS scores between the AC and PC groups. Previous studies in which patients' last follow-up mRS scores all increased have indicated a poor treatment outcome of PED, which could be due to the high average number of PEDs applied and the small sample sizes (5, 7–10).

## Limitations

This study has some limitations that should be noted. First, this was a retrospective, multi-center study, which means that the heterogeneity of the operator's operating preferences cannot be well quantified and counted. Second, the average follow-up time was short, and a longer follow-up time is needed to evaluate long-term efficacy. Third, a prospective, systematic study is needed to evaluate the safety and efficacy of FDD in the treatment of intracranial fusiform aneurysms.

## Conclusions

This study revealed that non-overlapping PED is safe and effective for the treatment of intracranial fusiform aneurysms. The long-term mRS score significantly improved after treatment. The occlusion rate of AC fusiform aneurysms was lower than that of PC fusiform aneurysms. The occlusion rate of fusiform aneurysms involving perforating vessels was low.

## Data Availability Statement

The original contributions presented in the study are included in the article/supplementary material, further inquiries can be directed to the corresponding author.

## Ethics Statement

The studies involving human participants were reviewed and approved by the Institutional Review Board (IRB) of Beijing Tiantan Hospital Affiliated to Capital Medical University (Ethical review No: KY288 2018-098-02). Written informed consent from the patients/participants legal guardian/next of kin was not required to participate in this study in accordance with the national legislation and the institutional requirements.

## Author Contributions

Conception and design: CX and PW. Acquisition of data: CX, PW, and BL. Analysis and interpretation of data: CX, LZ, and BL. Drafting the article: CX. Approving the final version of the manuscript on behalf of all authors: HS. Statistical analysis: CX and SX. Study supervision: HS and XY. All authors critically revised the article and reviewed the submitted version of manuscript.

## Funding

This study was funded by National Nature Science Foundation of China (Grant no. 82071309), National Natural Science Foundation of China (Grant no. 81901190), and Natural Science Foundation of Heilongjiang Province of China (Grant no. YQ2019H015).

## Conflict of Interest

The authors declare that the research was conducted in the absence of any commercial or financial relationships that could be construed as a potential conflict of interest.

## Publisher's Note

All claims expressed in this article are solely those of the authors and do not necessarily represent those of their affiliated organizations, or those of the publisher, the editors and the reviewers. Any product that may be evaluated in this article, or claim that may be made by its manufacturer, is not guaranteed or endorsed by the publisher.
